# Quality of Life in Patients with Chronic Pancreatitis after Frey’s Procedure at Tertiary Care Centre: An Observational Study

**DOI:** 10.31729/jnma.v63i2091.9225

**Published:** 2025-11-30

**Authors:** Nishef Adhikari, Gita Rana, Sampanna Pandey, Deepak Sharma, Ramesh Singh Bhandari, Paleswan Joshi Lakhey

**Affiliations:** 1 Department of Surgical Gastroenterology, Tribhuvan University Teaching Hospital, Kathmandu, Nepal

**Keywords:** *chronic*, *pain*, *pancreatitis*, *quality-of-life*, *surgery*

## Abstract

**Introduction:**

Approximately 90% of chronic pancreatitis patients experience chronic pain severely impairing quality of life. While medical treatment is usually the first approach, many patients ultimately require surgical intervention. Frey’s procedure provides technical advantages and favourable outcomes with a substantial reduction in chronic pain. This study aimed to assess its impact on pain relief and quality of life in a South Asian context.

**Methods:**

A prospective observational follow-up study was conducted in May and June 2025 after obtaining ethical approval. This study included patients who underwent Frey’s procedure for chronic pancreatitis in the Department of Surgical Gastroenterology. Quality of life was evaluated using the EORTC QLQ-C30 Version 3 questionnaire, while pain levels were assessed using the Izbicki pain score and the Visual Analogue Scale (VAS). Statistical analysis was performed using IBM SPSS version 26.

**Results:**

A total of 27 cases were enrolled in the study, with a median age of 36 (IQR: 23-46)years. There were 19 (70.37%) females. Non-alcoholic chronic pancreatitis was present in 16 (59.25%) cases and alcoholic in 11 (40.75%). The average postoperative hospital stay was 8.77±2.45 days. Sixteen (59.25%) cases had no complications, and 1 (3.70%) patient experienced a major complication. Postoperative VAS scores decreased from 79.26±24.95 to median of 0 (IQR: 0-20), and the median postoperative Izbicki pain score was 2.75(IQR: 0.25-11.50). Quality of life scores were favourable across all domains, with a Global Health score of 12.89±1.19 out of 14.

**Conclusions:**

There are favourable outcomes in different domains of health improving quality-of-life in patients undergoing Frey’s procedure for chronic pancreatitis with significant reduction in pain.

## INTRODUCTION

Approximately 90% of chronic pancreatitis (CP) patients experience chronic pain that restricts daily activities, limits physical and social roles, disrupts routine, and affects mental and emotional health effects. This severely impairs quality of life and often leads to hospitalizations and narcotics use.^1,2^ Chronic pancreatitis is a progressive inflammation of the pancreas characterized by persistent pain and reduced exocrine and endocrine functions.^3^ While alcohol is the primary cause in the West, India, having highest incidence globally, reports more tropical pancreatitis; likely due to genetic, metabolic, and nutritional factors.^4,5^

As CP is incurable, treatment focuses on pain relief and improving quality of life.^6^ Despite initial conservative treatment, 40-75% of CP patients eventually need surgery.^7^ Surgery is indicated for persistent pain and complications.^8,9^ Duodenum-preserving procedures, particularly Frey’s procedure are preferred for its technical simplicity and fewer anastomoses.^10-13^

Frey’s procedure provides excellent outcomes by reducing or eliminating pain and minimizing opioid use.^14^ This study aims to evaluate the quality of life in patients with CP post Frey’s procedure.

## METHODS

A prospective observational study was conducted in May and June 2025 following approval from the Institutional Review Committee. The primary objective was to assess the quality of life in patients with chronic pancreatitis who underwent Frey’s procedure in the Department of Surgical Gastroenterology. The study group comprised individuals who underwent Frey’s procedure at the center between December 16, 2021, and December 15, 2024, identified from prospectively maintained electronic medical records. A prospective follow-up of these patients was done with structured interviews at the outpatient department (OPD). To the patients residing at a significant geographical distance from the study centre, follow-up was conducted with telephone calls using a standardized interview protocol to ensure consistency in data collection. All surgeries were performed by consultant gastrointestinal (GI) and hepatopancreatobiliary (HPB) surgeons, and all patients completed a minimum of three months of postoperative follow-up. Informed consent was obtained in accordance with ethical guidelines. Patients who declined participation or were lost to follow-up, including those who missed scheduled visits or did not respond to telephone calls, were excluded. Individuals with less than three months of follow-up after Frey’s procedure were excluded to ensure an adequate period for quality-of-life assessment and pain scoring. Patients who underwent procedures other than Frey’s were also excluded.

Perioperative parameters were obtained from prospectively maintained electronic records. Postoperative complications were measured using Clavien-Dindo classification. Grade 3 or more were considered major complications, and lower grades were considered minor. During OPD follow-up and telephone calls, quality of life data were collected prospectively using the European Organisation for Research and Treatment of Cancer Quality of Life Questionnaire Core 30 (EORTC QLQ-C30) Version 3 and the Izbicki pain score.^15,16^ The EORTC QLQ-C30 is a validated instrument developed by the EORTC study group to assess quality of life across mental, social, emotional, and physical health domains. This includes 30 different questions to address those domains. Patients’ experience of individual questions as their problem is categorised as “No problem at all” with a score of 1, “A little problem” with 2, “Quite a bit” with 3, and “Very much” with 4, where a lower score reflects better experience. For this study, the 30 items were categorized into five domains: Physical Function Domain (PFD), Physical Domain (PD), Emotional Domain (ED), Social Domain (SD), and Global Health (GH). All questions in each domain were assessed with a scoring system as mentioned above, except that Global health was assessed with 2 questions addressing patients’ experience of overall health and quality of life, with very poor to excellent response scores ranging from 1 to 7. The higher the score, the better the function for the global health domain, whereas the lower the value, the better the function for the remaining domains. Izbicki pain score was calculated by measuring the frequency of pain attacks, requirement of analgesic medication, and time of disease-related inability to work. Patents were asked if they were having pain daily, several times a week, several times a month, several times a year, or none, and scores were allocated 10, 75, 50, 25, and 0, respectively. Depending upon the type of analgesic requirement, from paracetamol to morphine, a score was allocated from 1 to 100. Again, the time of disease-related inability to work was categorized as permanent, less than a year, less than a month, less than a week, or no restriction. Scores were allocated as 100, 75, 50, 25, and 0, respectively. Visual analogue scale score was also incorporated from 1 to 100, depending on the patient’s response. Finally, after the addition of all components, the score was averaged at 4. A score between 0-10 means no or minimal pain, and 50% or more reduction in the score after intervention was considered a significant clinical improvement.

Data were recorded and analyzed using IBM SPSS Statistics version 26.0 (IBM Corp., Armonk, NY, USA). Continuous variables were summarized as mean and median, while categorical variables were expressed as frequency and percentage.

## RESULTS

Thirty-one patients were identified to be followed during the study period; four of them were lost to follow-up. Twenty-seven cases were included in the study. There were 11 (40.74%) patients who were followed up at OPD, and 16 (59.26%) patients with telephone calls. The mean post-operative hospital stay was 8.77±2.45 days while the media follow up period for patients was 19 (IQR: 6-28) months.

The median age of the patients was 36 (IQR: 23-46) years and 19 (70.37%) patients were female. There were 11 (40.75%) patients with alcoholic chronic pancreatitis and 16 (59.25%) patients had non-alcoholic causes. Postoperative complications, as classified by the Clavien-Dindo system which showed 16 (59.26%) cases experienced no complications. Minor complications were reported in 5 (18.52%) patients as Grade I, and 5 (18.52%) as Grade II. Only 1 (3.70%) patient experienced a Grade IIIb complication, requiring intervention without general anaesthesia. This patient developed hematemesis and melena on the seventh postoperative day, features of intraluminal bleed, with a significant drop in haemoglobin (6.6g/dL). Angiography on computed tomography (CT) showed contrast blush from the superior pancreaticoduodenal artery, for which Gelfoam angioembolization was done. There was 0 (0%) in-hospital mortality (Clavien-Dindo V=0)(Table 1).

The mean preoperative VAS score was 79.26±24.95, while the median score during follow-up after surgery was 0 (IQR: 0-20). The mean reduction in pain score was 70.37±26.53. During follow-up, the median Izbicki pain score was 2.75 (IQR: 0.25-11.5), with scores ranging from 0 to 22.75 (Table 2).

**Table 1:** Characteristics of patients who had undergone Frey’s procedure (n=27).

Variables	n(%)
**Gender**	
Female	19(70.37)
Male	8(29.63)
**Cause of chronic pancreatitis**	
Non-alcoholic	16(59.26)
Alcoholic	11(40.74)
**Clavien-Dindo classification**	
**Minor complications**	
Grade I	5(18.52)
Grade II	5(18.52)
**Major complications**	
Grade IIIa	1(3.70)
Grade IIIb	0(0.00)
Grade IVa	0(0.00)
Grade IVb	0(0.00)
Grade V (Mortality)	0(0.00)

**Table 2:** Pain parameters before and after Frey’s procedure (n=27).

Variables	Score
Preoperative VAS Score	79.26±24.95
Median VAS Score during follow-up	0(0-20)
Mean reduction in VAS Score	70.37±26.53
Median Izbicki pain score during follow-up	2.75(0.25-11.50)

**Table 3:** Quality of life parameters after Frey’s procedure (n=27).

Variables	Mean ± SD/Median	Best & Worst Possible Score
Physical Function Domain	9.71±1.03	9 & 36^*^
Physical Domain	11.33±1.79	10 & 40^*^
Emotional Domain	6.44±1.97	6 & 24^*^
Social Domain	3.71±1.46	3 & 12^*^
Global Health	12.89±1.19	14 & 2^**^

* Lesser the value, better the function

** Higher the value, better the function

Quality of life assessed using the EORTC QLQ-C30 V3 questionnaire showed generally favorable outcomes across most domains following Frey’s procedure. The Physical Function Domain had a mean score of 9.71±1.03, the Physical Domain had 11.33±1.79, the Emotional Domain had 6.44±1.97, and the Social Domain had 3.71±1.46. The Global Health Status had a mean score of 12.89±1.19 (maximum possible: 14) (Table 3).

## DISCUSSION

Chronic pancreatitis is a debilitating, incurable disease that significantly affects the physical, emotional, and social wellbeing of patients. Pain is the most prominent and distressing symptom, frequently resulting in reduced quality of life and increased healthcare utilization. Persistent pain and repeated hospital admissions also contribute to psychological distress, such as anxiety and depression, which further impair overall functioning and social engagement.^17^

Conservative management and endoscopic interventions are frequently used as initial treatments; however, their long-term effectiveness is often limited, and many patients eventually require surgical intervention. Surgical procedures that combine ductal decompression with resection of the inflammatory pancreatic head mass have demonstrated more sustained pain relief and improved functional recovery. Frey’s procedure, in particular, offers a balance between decompression and limited resection while preserving pancreatic parenchyma.^18^

This study explored the quality of life in patients with CP who had undergone Frey’s procedure in our setting. Our patients were younger, with a median age of 36 (23-46) years. The majority of our patients had non-alcoholic chronic pancreatitis (59.26%). Seventy-five percent of males had alcoholic chronic pancreatitis, whereas 73.68% of females had non-alcoholic cause. There was a significant reduction in pain severity, with the mean VAS score decreasing from 79.26±24.95 preoperatively to a median score of 0 (0-20) postoperatively. The median postoperative Izbicki pain score of 2.75 (0.25-11.50) indicates a complete or near-complete response in the majority of patients. Only seven of our patients had an Izbicki score above ten, still having more than 50% reduction in VAS, which is considered a significant clinical improvement. There was a substantial improvement in postoperative quality of life. The Physical Function and Physical Domains had low mean scores (9.71±1.03 and 11.33±1.79, respectively), indicating good functional capacity and minimal symptom burden. Emotional and social functioning were also preserved, and the Global Health score was high (mean: 12.89±1.19 out of 14), reflecting overall satisfaction and wellbeing after surgery.

Demographics of our patients align with the Indian subcontinent population, where more cases of CP belong to the younger age group. Prakash VB et al. in 2025, in a hospital-based study from India, identified 75% of the cases of CP were at 19-45 years of age.^4^ Our findings also conform to South Asian epidemiology, where idiopathic or non-alcoholic tropical pancreatitis predominates. Nutritional factors, genetic predisposition, and environmental exposures are held to be responsible for the earlier presentation and non-alcoholic or idiopathic cause.^4,5^ We also noticed a significantly higher proportion of male gender having alcoholic etiology in comparison to females, as it could be the cause of known alcohol consuming behavioral risk factors.^5^

The present study demonstrates substantial improvements in pain outcomes. The “ESCAPE Randomized Clinical Trial”, published in 2020, showed complete or partial relief of pain among 58% of the patients at the end of 18 months of follow-up after surgical intervention for CP.^19^ The follow-up period of our patients after Frey’s procedure was comparable, with a median period of 19 (6-28) months, where 20 (74.07%) patients had a complete or near complete response in terms of pain relief as demonstrated by the median postoperative Izbicki pain score. Ray S et al. in 2021 analysed 138 cases of chronic pancreatitis who had undergone Frey’s procedure, and showed that an even better outcome can be achieved with 84% of complete pain relief.^20^

The categorization of different domains of quality-of-life, previously applied by Rath S et al. in a 2016 study, provided a practical framework for domain-specific analysis of QoL outcomes in surgical cohorts.^21^ Our findings are consistent with previous literature reporting significant QoL improvements following Frey’s procedure, particularly when surgery is performed before irreversible pancreatic damage develops, as demonstrated by a systematic review by Ashfaq A et al. in 2024, which noted a significant achievement in pain control and overall quality of life with a significant reduction in analgesic requirement.^22^ Quality of life is also achieved through favourable perioperative outcomes with lower mortality and morbidities. This study further corroborates the evidence that Frey’s procedure enhances quality of life with minimal postoperative complications. The majority of our patients had either no complications or minor complications graded as Clavien-Dindo Grade I and II. Those minor complications that typically occurred were self-limiting in nature, such as transient fever, superficial wound infection, or delayed bowel function, which resolved with conservative management. Only one patient in the entire group experienced a Grade IIIa complication, which required intervention but without the need for reoperation. The mean in-hospital stay for our series was 8.77±2.45 days, consistent with other centers that perform this procedure. Ray S et al., in a similar study in 2021, showed a median hospital stay of 9 days with postoperative complications in 31% cases, with no mortality.^20^ Complications were experienced by 40.72% of our cases, but all were with minor complications except for one (3.7%). We also experienced no mortality in our study.

When compared to published literature, our outcomes are consistent with international experience, where the quality of life in patients with CP is improved with surgery, particularly with drainage and limited resection procedures like Frey’s procedure. Postoperative quality oflife is primarily determined by pain relief, and various studies mentioned above have reported sustained reduction in pain scores and the requirement of analgesics, especially opioids, after surgery. Improvement in nutritional status, adequate weight gain, and betterment in physical and emotional well-being have been significantly demonstrated. Nutritional improvement and significant weight gain also reflect the preservation of pancreatic function. Superior long-term outcomes are achieved before irreversible pancreatic damage and psychosocial deterioration.^23^ Productivity improvement, reflected by physical well-being and social functioning, reflects the positive outcomes after surgery, restoring daily living activities.

This study contributes valuable data in support of improving the of life among cases of CP who had undergone Frey’s procedure. With emphasis on patients that are different from the Western population, our findings contribute to the evidence base and underscore the necessity for optimization of management strategies according to local epidemiologic patterns. Our patients perceived substantial and sustained relief of pain, which is the key therapeutic goal in chronic pancreatitis. Also noteworthy, postoperative measurements recorded substantial quality-of-life improvement, supporting the fact that surgical correction can restore function as well as enhance functioning for patients who have suffered debilitating symptoms for many years. These observations attest that with an efficacious and reasonable treatment modality, significant achievement can be gained in terms of quality of life in this incurable disease.

The strengths of our research are a prospectively observed group of patients, clinically corroborated QoL questionnaires, and relatively long follow-up. However, there are a few limitations to be noted. First, the sample size was limited (n = 27), limiting the detection of differences between subgroups. Second, the lack of a control group (e.g., patients undergoing other surgery or being treated conservatively) restricts conclusions in comparative terms. Lastly, despite efforts at minimizing recall bias with OPD and telephone follow-up, patient-reported outcomes are subjective and time-dependent.

## CONCLUSIONS

We conclude Frey’s procedure as a safe and effective surgical option for long-term pain control and functional recovery in appropriately selected patients with chronic pancreatitis as favourable outcomes across physical, emotional, and social domains, with minimal postoperative dysfunction and high global health perception were observed.

## Data Availability

The data are available from the corresponding author upon reasonable request.
